# Risk factors associated with the co-occurrence of severe pain and sleep disturbance in oncology outpatients receiving chemotherapy

**DOI:** 10.1007/s00520-025-10151-2

**Published:** 2025-11-19

**Authors:** Aean Peattie, Sueann Mark, Astrid Block, Bruce A. Cooper, Steven M. Paul, Marilyn J. Hammer, Frances Cartwright, Yvette P. Conley, Jon D. Levine, Christine Miaskowski

**Affiliations:** 1 School of Nursing, University of California, 490 Illinois Street, Floor 12, San Francisco, CA 94143-0610 USA; 2 Phyllis F. Cantor Center, Dana Farber Cancer Institute, Boston, MA USA; 3 Mount Sinai Medical Center, New York, NY USA; 4 School of Nursing, University of Pittsburgh, Pittsburgh, PA USA; 5 School of Medicine, University of California, San Francisco, CA USA

**Keywords:** Cancer, Chemotherapy, Insomnia, Multimorbidity, Pain, Pain interference, Pain qualities, Sleep disturbance

## Abstract

**Purpose:**

Study purposes were to identify subgroups of patients with distinct co-occurring pain AND sleep disturbance profiles and evaluate for differences in demographic, clinical, pain, and sleep characteristics between the subgroups.

**Methods:**

Oncology outpatients receiving chemotherapy (*n* = 972) completed self-report questionnaires on various demographic and clinical characteristics. Pain and sleep disturbance were assessed six times over two cycles of chemotherapy, using the Brief Pain Inventory and the General Sleep Disturbance Scale, respectively. A joint latent profile analysis was performed using the six ratings of worst pain severity and sleep disturbance. Parametric and non-parametric tests were used to evaluate for differences in modifiable and non-modifiable risk factors between the profiles.

**Results:**

Two subgroups of patients with distinct joint pain and sleep disturbance profiles were identified (i.e., Moderate Pain and Sleep Disturbance (Both Moderate, 53.4%) and Severe Pain and Sleep Disturbance (Both Severe, 46.6%)). Compared to the Both Moderate class, patients in Both Severe class were younger, female, had lower level of education, were unemployed, and had a lower annual income. In addition, they had a higher comorbidity burden and a lower functional status. The Both Severe class had problems with sleep initiation and maintenance.

**Conclusions:**

A significant proportion of patients receiving chemotherapy experience the co-occurrence of severe pain and sleep disturbance. Oncology clinicians need to work with primary care providers to optimize the management of these two symptoms.

## Introduction

Pain is one of the most common symptoms reported by patients undergoing cancer treatment [1]. While moderate to severe chronic pain affects about 20% of the general population [2], a recent meta-analysis determined that 30.6% of cancer patients undergoing active treatment report moderate to severe pain [1]. Although this rate represents improvements over previous years [3], a significant proportion of cancer patients continue to experience pain. Uncontrolled pain interferes with patients’ ability to perform routine activities of daily living and has a negative impact on their quality of life (QOL) [4]. Equally important, sleep disturbance occurs in almost 60% of oncology patients [5], almost twice the rate observed in the general population [6]. Inadequate sleep has numerous negative consequences on health status [7], QOL [8], and survival [9].

Pain and sleep disturbance frequently co-occur in cancer patients [10–13]. Research in the general population suggests that sleep disturbance drives this association, because sleep disorders more frequently precede and exacerbate pain than vice versa [14]. However, evidence from patients undergoing cancer treatment remains inconclusive. In some studies [15, 16], pain ranked relatively low as a cause for sleep disturbance compared to, for example, nocturia, even in samples of oncology patients with high pain prevalence rates. In contrast, in a study of women with metastatic breast cancer [17], patients with higher levels of pain at enrollment reported increasing difficulty falling asleep over the course of the study even if their pain levels did not increase over time. This finding led the authors to suggest that the higher pain scores at enrollment were at least in part, driving the increasing sleep onset latency. However, other measures of sleep disturbances (i.e., sleep duration, waking after sleep onset) were not associated with pain severity.

Previous research from our team demonstrated a large amount of inter-individual variability in oncology patients’ experiences with pain [4] and sleep disturbance [18]. Using latent profile analysis (LPA) to identify distinct worst pain profiles in oncology patients receiving chemotherapy [4], three classes (i.e., Low, Moderate, Severe) were found. Patients in the Severe Worst Pain class were more likely to be female, single, and unemployed, have a lower annual income, a higher number of comorbidities, and a lower functional status. In addition, they were more likely to have both cancer and non-cancer pain, a higher frequency of pain, and worse pain interference scores. In the LPA of sleep disturbance [18], three distinct sleep disturbance profiles (Low, High, Very High) were identified. Compared to the Low class, patients in the High and Very High classes were more likely to be younger, female, and unemployed. In addition, these patients had a higher comorbidity burden and a lower functional status. These two studies support additional investigations of inter-individual variability in patients’ experiences with both pain and sleep disturbance, as well as risk factors associated with the co-occurrence of both symptoms.

Therefore, given the paucity of longitudinal studies on the co-occurrence of pain and sleep disturbance in oncology outpatients receiving chemotherapy, the purposes of this study were to identify subgroups of patients with distinct co-occurring pain AND sleep disturbance profiles and evaluate for differences in demographic, clinical, pain, and sleep characteristics between the subgroups. The risk factors identified in the current study will be compared to the findings from the single symptom LPAs [4, 18]. These findings can be used to initiate timely referrals and implement targeted interventions to decrease patients’ symptom burden.

## Methods

### Patients and settings

This study is part of a larger, longitudinal study of the symptom experience of outpatients receiving chemotherapy [19]. Eligible patients were ≥ 18 years of age; had a diagnosis of breast, gastrointestinal, gynecological, or lung cancer; had received chemotherapy within the preceding four weeks; were scheduled to receive at least two additional cycles of chemotherapy; were able to read, write, and understand English; and gave written informed consent. Patients were recruited from two Comprehensive Cancer Centers, one Veteran’s Affairs hospital, and four community-based oncology programs. The major reason for refusal was being overwhelmed with their cancer treatment.

### Study procedures

The study was approved by the Institutional Review Board at each of the study sites. Of the 2234 patients approached during their first or second cycle of chemotherapy, 1343 consented to participate. Patients completed questionnaires, six times over two chemotherapy cycles (i.e., prior to chemotherapy administration (assessments 1 and 4); approximately 1 week after chemotherapy administration (assessments 2 and 5); and approximately 2 weeks after chemotherapy administration (assessments 3 and 6)). Medical records were reviewed for disease and treatment information. Of the 1343 patients, 972 reported both pain and sleep disturbance and were evaluated in this analysis.

### Instruments

#### Demographic and clinical measures

Patients completed a demographic questionnaire, Karnofsky Performance Status (KPS) scale [20], Self-Administered Comorbidity Questionnaire (SCQ) [21], Alcohol Use Disorders Identification Test (AUDIT) [22], and a smoking history questionnaire. Medical records were reviewed for disease and treatment information. Toxicity of the chemotherapy regimen was evaluated using the MAX2 score [23].

#### Pain measure

Worst pain severity was assessed using the Brief Pain Inventory (BPI) [24]. Patients were asked to indicate whether they were generally bothered by pain (yes/no). If they were generally bothered by pain, they indicated if they had non-cancer pain, cancer pain, or both types of pain. Then, patients rated their worst pain severity in the past 24 h using a 0 (no pain) to 10 (worst pain imaginable) numeric rating scale (NRS). Additional items that were evaluated included: current and average pain intensity; number of days per week that pain interfered with mood and/or activities; number of hours per day in pain; number of pain locations; percentage pain relief from pain medications; causes of non-cancer pain; duration and frequency of pain; analgesic intake; pain qualities; and pain interference with eight activities. For the pain interference items, a total mean score was computed.

#### Sleep disturbance measure

The General Sleep Disturbance Scale (GSDS) consists of 21 items designed to assess various aspects of sleep disturbance (i.e., quality, quantity, onset latency, mid and early awakenings, sleep medications, daytime sleepines) [25]. Each item was rated on a 0 (never) to 7 (everyday) NRS. The GSDS total score ranges from 0 (no disturbance) to 147 (extreme sleep disturbance). Each mean subscale score ranges from 0 to 7. Subscale scores of ≥ 3 and a GSDS total score of ≥ 43 indicate a significant level of sleep disturbance that warrants clinical evaluation and management [26]. The GSDS has well-established validity and reliability. Cronbach’s alpha for the GSDS total score was 0.83.

### Data analysis

Latent profile analysis (LPA) was used to identify subgroups of patients with distinct joint pain AND sleep disturbance profiles. Using Mplus version 8.4 [27], this LPA was done with the combined set of variables over time (i.e., using the worst pain scores for the patients who reported pain AND the GSDS total scores obtained for each of the six assessments in a single LPA)*.* This approach provides a joint profile description of these two symptoms over time.

Estimation was carried out with full information maximum likelihood (FIML) [28, 29] with standard errors and a chi-square test that are robust to non-normality and non-independence of observations. Model fit was evaluated to identify the solution that best characterized the observed latent class structure with the Bayesian Information Criterion, Vuong-Lo-Mendell-Rubin likelihood ratio test, entropy, and latent class percentages that were large enough to be reliable [30]. Model estimation was carried out with from 800 to 2200 random starts to ensure that the best fitting model was selected multiple times and was not due to a local maximum.

An additional advantage of using FIML for LPA estimation is that missing data are accommodated using FIML with the Expectation–Maximization (EM) algorithm [28, 31]. This method provides unbiased parameter estimates if the missingness is missing at random (MAR). This assumption is reasonable for the proposed study, because missingness should be associated either with previous measures of the outcome or for pain associated with sleep disturbance and vice versa (missing at random, covariate-dependent missingness). Some missingness might be “missing completely at random” for reasons that have nothing to do with the study or the outcome variables (e.g., an assessment might be missing if a patient is unable to attend a scheduled treatment day due to transportation problems).

Six measures of pain and six of sleep disturbance, reported by 972 patients, were evaluated. A total of 201 missing data patterns were identified. Sample size for the six measures of pain ranged from 722 at assessment one to 567 at assessment six. The range of cases with measures of sleep disturbance ranged from 934 at assessment one to 789 at assessment six. The range for pairwise covariance coverage for the 12 measures was from a low of 0.441 for paired pain scores to a high of 0.929 for paired sleep disturbance scores. Of course, consistent with the smaller sample size for worst pain measures compared to sleep disturbance measures, the covariance coverage was lower for the pain measures. However, given the large sample size, Mplus FIML with the EM Algorithm provides robust estimates of the LPA mean profiles and standard errors.

In order to incorporate expected correlations among the repeated measures of the same variable and cross-correlations of the series of the two variables (i.e., worst pain and GSDS scores), covariance parameters among measures at the same occasion and those that were one or two occasions apart were included. Covariances of each variable with the other at the same assessments were included in the model and autoregressive covariances were estimated with a lag of two with the same measures and with a lag of one for each variable’s series with the other variable. The covariance structure was limited to a lag of two to accommodate the expected reduction in the correlations that would be introduced by two chemotherapy cycles within each set of three measurement occasions and to reduce model complexity [32].

Descriptive statistics and frequency distributions were generated for sample characteristics at enrollment using IBM SPSS Statistics version 29 (IBM Corporation, Armonk, NY). Differences between the latent classes in demographic, clinical, pain, and sleep disturbance characteristics were evaluated using independent Student’s *t*-tests, Fisher’s exact tests, chi-square tests, or Mann–Whitney *U* tests. A *p*-value of < 0.05 was considered statistically significant.

## Results

### Latent profile analysis

**Fig. 1:**
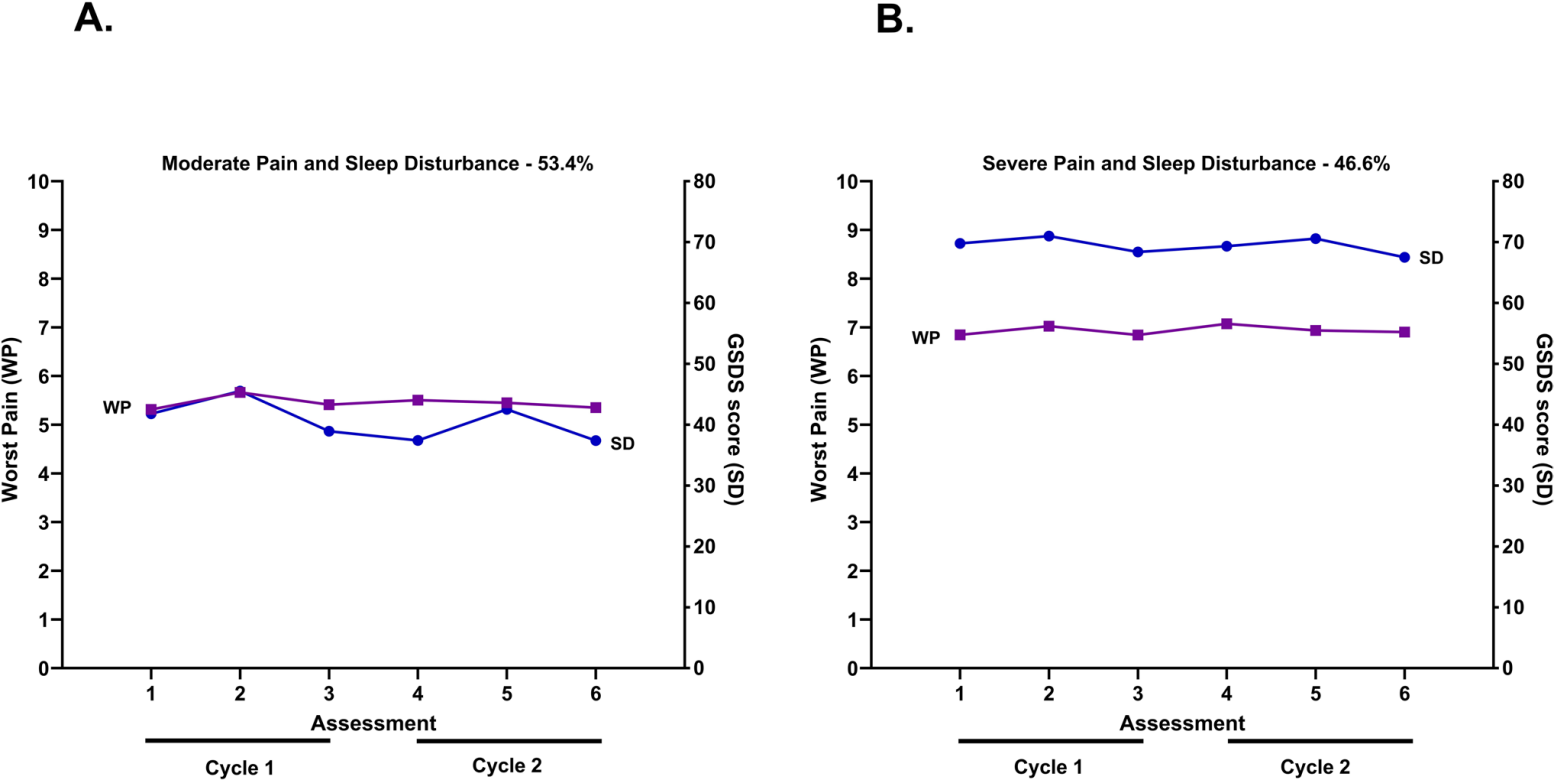
Trajectories of worst pain (WP—left *y*-axis) and sleep disturbance (SD—right *y*-axis) for the two latent classes. The numbers on the *x*-axis indicated the assessments of worst pain (i.e., 0 to 10 numeric rating scale) and sleep disturbance (i.e., General Sleep Disturbance (GSDS) scores) that were done prior to the administration of chemotherapy (i.e., assessments 1 and 4); in the week following the administration of chemotherapy (i.e., assessments 2 and 5); and 2 weeks after the administration of chemotherapy (i.e., assessments 3 and 6). Assessments 1, 2, and 3 represent the first cycle of chemotherapy. Assessments 4, 5, and 6 represent the second cycle of chemotherapy

The rationale for the two-class solution is presented in Table 1. As shown in Fig. 1, of the 972 patients who reported both pain and sleep disturbance, 53.4% were in the Moderate Pain and Sleep Disturbance class (i.e., Both Moderate) and 46.6% were in the Severe Pain and Sleep Disturbance class (i.e., Both Severe). For the Both Moderate class, while the pain scores remained relatively stable across the six assessments, sleep disturbance scores increased at assessments two and five (i.e., following the administration of chemotherapy). For the Both Severe class, pain and sleep disturbance score remained relatively stable over time.

**Table 1:** Latent profile solutions and fit indices for one through three classes for worst pain and sleep disturbance

Model	LL	AIC	BIC	Entropy	VLMR
1 Class	− 28,982.27	58,080.53	58,363.53	n/a	n/a
2 Class^a^	− 28,605.68	57,353.36	57,699.80	0.75	753.17^‡^
3 Class	− 28,436.49	57,040.97	57,450.84	0.78	ns

Baseline entropy and VLMR are not applicable for the one-class solution

^‡^*p* <.00005

^a^The 2-class solution was selected because the BIC for that solution was lower than the BIC for the baseline (1-class) solution. In addition, the VLMR was significant for the 2-class solution, indicating that two classes fit the data better than one classes. Although the BIC was smaller for the 3-class than for the 2-class solution, the VLMR was not significant for the 3-class solution, indicating that too many classes were extracted

Abbreviations: *AIC*, Akaike’s Information Criterion; *BIC*, Bayesian Information Criterion; *LL*, log-likelihood; *n/a*, not applicable; *ns*, not significant; *VLMR*, Vuong-Lo-Mendell-Rubin likelihood ratio test for the K vs. K-1 model

### Demographic and clinical characteristics

Compared to the Both Moderate class, patients in the Both Severe class were younger, had a lower level of education, and were more likely to be female, unemployed, not married or partnered, and living alone. In addition, they had a lower annual income and were more likely to have child care responsibilities, less likely to exercise on a regular basis, more likely to self-report being Hispanic, Mixed, or other race/ethnicity, and less likely to self-report being an Asian or Pacific Islander (Table 2).

**Table 2:** Differences in demographic and clinical characteristics between the pain and sleep disturbance classes

Characteristics	Moderate pain and sleep disturbance (1) 53.4% (*n* = 519)	Severe pain and sleep disturbance (2) 46.6% (*n* = 453)	Statistics
	Mean (SD)	Mean (SD)	
Age (years)	58.5 (12.5)	54.8 (12.3)	*t* = 4.63, *p* <.001 1 > 2
Education (years)	16.3 (3.0)	15.7 (2.9)	*t* = 2.71, *p* =.007 1 > 2
Body mass index (kg/m^2^)	26.0 (5.3)	26.9 (6.2)	*t* = − 2.33, *p* =.020 1 < 2
Alcohol Use Disorders Identification Test score	2.8 (2.4)	3.1 (2.9)	*t* = − 1.47, *p* =.143
Karnofsky Performance Status score	81.1 (11.9)	74.7 (12.1)	*t* = 8.08, *p* <.001 1 > 2
Number of comorbid conditions	2.4 (1.5)	2.8 (1.5)	*t* = − 3.77, *p* <.001 1 < 2
Self-administered Comorbidity Questionnaire score	5.4 (3.0)	6.6 (3.6)	*t* = − 5.61, *p* <.001 1 < 2
Time since diagnosis (years)	2.3 (4.2)	1.9 (3.9)	*U*, *p* =.636
Time since diagnosis (years, median)	0.44	0.42	
Number of prior cancer treatments	1.7 (1.6)	1.7 (1.5)	*t* = − 0.13, *p* =.893
Number of metastatic sites including lymph node involvement^a^	1.4 (1.3)	1.2 (1.3)	*t* = 1.78, *p* =.076
Number of metastatic sites excluding lymph node involvement	0.87 (1.1)	0.79 (1.1)	*t* = 1.24, *p* =.214
MAX2 score	0.17 (0.08)	0.18 (0.08)	*t* = − 1.59, *p* =.111
	% (*n*)	% (*n*)	
Gender (% female)	76.7 (398)	83.9 (380)	FE, *p* =.006 1 < 2
Self-reported ethnicity			*Χ*^2^ = 12.46, *p* =.006
White	68.2 (347)	70.1 (314)	NS
Asian or Pacific Islander	15.3 (78)	9.8 (44)	1 > 2
Black	8.1 (41)	6.5 (29)	NS
Hispanic, mixed, or other	8.4 (43)	13.6 (61)	1 < 2
Married or partnered (% yes)	67.2 (344)	57.2 (254)	FE, *p* =.002 1 > 2
Lives alone (% yes)	18.1 (93)	27.4 (122)	FE, *p* <.001 1 < 2
Currently employed (% yes)	36.1 (186)	28.1 (126)	FE, *p* =.009 1 > 2
Annual household income			*U*, *p* =.002
Less than $30,000^+^	14.4 (66)	29.7 (123)	1 > 2
$30,000 to $70,000	26.5 (121)	18.6 (77)	
$70,000 to $100,000	17.7 (81)	13.8 (57)	
Greater than $100,000	41.4 (189)	37.9 (157)	
Child care responsibilities (% yes)	18.8 (95)	25.8 (115)	FE, *p* =.012 1 < 2
Elder care responsibilities (% yes)	9.1 (42)	8.1 (34)	FE, *p* =.632
Current or past history of smoking	37.7 (193)	36.9 (164)	FE, *p* =.841
Exercise on a regular basis (% yes)	71.6 (363)	64.0 (283)	FE, *p* =.015 1 > 2
Specific comorbid conditions (% yes)			
Heart disease	5.6 (29)	7.9 (36)	FE, *p* =.157
High blood pressure	30.3 (157)	31.1 (141)	FE, *p* =.781
Lung disease	11.8 (61)	12.6 (57)	FE, *p* =.695
Diabetes	10.0 (52)	9.7 (44)	FE, *p* =.914
Ulcer or stomach disease	4.2 (22)	7.1 (32)	FE, *p* =.067
Kidney disease	1.3 (7)	2.0 (9)	FE, *p* =.460
Liver disease	8.1 (42)	5.5 (25)	FE, *p* =.128
Anemia or blood disease	11.8 (61)	15.9 (72)	FE, *p* =.062
Depression	14.5 (75)	31.6 (143)	FE, *p* <.001 1 < 2
Osteoarthritis	14.1 (73)	15.2 (69)	FE, *p* =.649
Back pain	28.3 (147)	38.0 (172)	FE, *p* =.002 1 < 2
Rheumatoid arthritis	4.2 (22)	4.0 (18)	FE, *p* =.873
Cancer diagnosis			*Χ*^2^ = 2.49, *p* =.477
Breast cancer	38.5 (200)	43.5 (197)	
Gastrointestinal cancer	30.1 (156)	27.4 (124)	
Gynecological cancer	19.5 (101)	18.3 (83)	
Lung cancer	11.9 (62)	10.8 (49)	
Prior cancer treatment			*Χ*^2^ = 3.16, *p* =.368
No prior treatment	25.0 (125)	21.3 (95)	
Only surgery, CTX, or RT	40.7 (204)	42.0 (187)	
Surgery and CTX, or surgery and RT, or CTX and RT	22.0 (110)	21.1 (94)	
Surgery and CTX and RT	12.4 (62)	15.5 (69)	
Metastatic sites			*Χ*^2^ = 4.81, *p* =.186
No metastasis	28.5 (147)	34.8 (155)	
Only lymph node metastasis	22.7 (117)	21.1 (94)	
Only metastatic disease in other sites	22.3 (115)	21.5 (96)	
Metastatic disease in lymph nodes and other sites	26.6 (137)	22.6 (101)	
CTX regimen			*Χ*^2^ = 9.47, *p* =.009
Only CTX	64.6 (330)	73.8 (329)	1 < 2
Only targeted therapy	3.7 (19)	2.5 (11)	NS
Both CTX and targeted therapy	31.7 (162)	23.8 (106)	1 > 2
Cycle length			*U*, *p* =.765
14-day cycle	40.7 (210)	40.9 (182)	
21-day cycle	51.0 (263)	52.1 (232)	
28-day cycle	8.3 (43)	7.0 (31)	
Emetogenicity of the CTX regimen			*U*, *p* =.614
Minimal/low	22.5 (116)	20.0 (89)	
Moderate	59.7 (308)	62.8 (280)	
High	17.8 (92)	17.3 (77)	
Antiemetic regimen			*Χ*^2^ = 4.12, *p* =.249
None	7.9 (40)	6.4 (28)	
Steroid alone or serotonin receptor antagonist alone	20.4 (103)	22.0 (96)	
Serotonin receptor antagonist and steroid	49.6 (250)	45.0 (196)	
NK-1 receptor antagonist and two other antiemetics	22.0 (111)	26.6 (116)	

^a^Total number of metastatic sites evaluated was 9

^+^Reference group

Abbreviations: *CTX*, chemotherapy; *FE*, Fisher’s exact test; *kg*, kilograms; *m*^*2*^, meters squared; *NK-1*, neurokinin-1; *NS*, not significant; *RT*, radiation therapy; *SD*, standard deviation; *U*, Mann–Whitney *U* test; *Χ*^*2*^, chi-square test

In terms of clinical characteristics, the Both Severe class had a lower KPS score, higher number of comorbid conditions, higher SCQ score, and higher body mass index (BMI). In addition, they were more likely to self-report diagnoses of depression and back pain, and more likely to have received only chemotherapy.

### Pain characteristics

Compared to the Both Moderate class, patients in the Both Severe class reported higher scores for current pain, average pain, worst pain, number of days per week in pain, number of hours per day in pain, number of pain locations, as well as the length of time in cancer and non-cancer pain, and the frequency of pain (Table 3). In addition, they reported higher scores for all of the pain interference items and the total pain interference score; had lower satisfaction scores; and were more likely to have taken analgesics in the past week. In terms of causes of pain, patients in the Both Severe class were more likely to report the occurrence of both cancer and non-cancer pain. For all of the pain qualities except numb, the Both Severe class reported higher occurrence rates.

**Table 3:** Differences in pain characteristics between the joint pain and sleep disturbance classes at enrollment

Characteristics	Moderate pain and sleep disturbance 53.4% (*n* = 519)	Severe pain and sleep disturbance^a^ 46.6% (*n* = 453)	Statistics
	Mean (SD)	Mean (SD)	
Pain now	1.4 (1.7)	2.4 (2.3)	*t* = − 6.85, *p* <.001 1 < 2
Average pain	2.6 (1.8)	3.6 (2.1)	*t* = − 6.76, *p* <.001 1 < 2
Worst pain	5.4 (2.5)	6.9 (2.3)	*t* = − 8.75, *p* <.001 1 < 2
Number of days per week in pain	2.4 (2.4)	3.5 (2.4)	*t* = − 6.84, *p* <.001 1 < 2
Number of hours per day in pain	6.4 (7.2)	9.9 (8.6)	*t* = − 6.13, *p* <.001 1 < 2
Number of pain locations	7.2 (6.4)	9.9 (8.9)	*t* = − 4.96, *p* <.001 1 < 2
Percentage of relief from pain medication	67.4 (31.5)	67.5 (26.5)	*t* = − 0.31, *p* =.975
Satisfaction with pain management	7.5 (2.4)	6.9 (2.6)	*t* = 3.60, *p* <.001 1 > 2
Pain interference			
General activity	2.6 (2.7)	3.9 (3.0)	*t* = − 6.28, *p* <.001 1 < 2
Mood	2.2 (2.5)	4.0 (2.8)	*t* = − 9.10, *p* <.001 1 < 2
Walking ability	2.6 (3.0)	3.6 (3.2)	*t* = − 4.58, *p* <.001 1 < 2
Normal work	2.9 (3.0)	4.3 (3.2)	*t* = − 6.51, *p* <.001 1 < 2
vRelations with other people	1.5 (2.3)	2.9 (2.8)	*t* = − 7.82, *p* <.001 1 < 2
Sleep	2.4 (2.6)	4.7 (3.0)	*t* = − 11.71, *p* <.001 1 < 2
Enjoyment of life	2.7 (2.9)	4.3 (3.0)	*t* = − 7.84, *p* <.001 1 < 2
Sexual activity	2.5 (3.5)	4.2 (4.1)	*t* = − 6.19, *p* <.001 1 < 2
Mean pain interference score	2.4 (2.2)	4.0 (2.6)	*t* = − 9.26, *p* <.001 1 < 2
	% (*n*)	% (*n*)	
Type of pain			*Χ*^2^ = 26.30, *p* <.001
Only noncancer pain	38.1 (172)	32.1 (132)	NS
Only cancer pain	26.2 (118)	15.8 (62)	1 > 2
Both cancer and noncancer pain	35.7 (161)	52.1 (214)	1 < 2
Causes of non-cancer pain (% yes)			
Headache	26.9 (75)	41.2 (114)	FE, *p* <.001 1 < 2
Low back pain	38.7 (108)	50.9 (141)	FE, *p* =.005 1 < 2
Fibromyalgia	2.5 (7)	4.7 (13)	FE, *p* =.180
Diabetic neuropathy	3.9 (11)	4.0 (11)	FE, *p* = 1.000
Arthritis	25.8 (72)	27.4 (76)	FE, *p* =.701
Other	44.2 (123)	41.7 (115)	FE, *p* =.549
Length of time with noncancer pain			
Less than 1 month	24.8 (60)	16.0 (41)	*U*, *p* =.001
2 to 6 months	16.1 (39)	11.3 (29)	1 < 2
Greater than 6 months	59.1 (143)	72.7 (186)	
Length of time with cancer pain			
Less than 1 month	43.0 (139)	30.7 (104)	*U*, *p* =.007
2 to 6 months	33.1 (107)	42.2 (143)	1 < 2
Greater than 6 months	23.8 (77)	27.1 (92)	
Frequency of pain			
1 to 4 times per month	24.2 (98)	18.0 (69)	*U*, *p* <.001
Several times per week	22.5 (91)	19.5 (75)	1 < 2
Multiple times per day	42.5 (172)	40.1 (154)	
Continuously	10.9 (44)	22.4 (86)	
Took pain medication in the last week (% yes)	54.4 (233)	71.7 (286)	FE, *p* <.001 1 < 2
Pain qualities (% yes)			
Aching	74.9 (314)	84.5 (333)	FE, *p* <.001 1 < 2
Throbbing	30.8 (128)	49.5 (194)	FE, *p* <.001 1 < 2
Shooting	26.3 (109)	39.3 (155)	FE, *p* <.001 1 < 2
Stabbing	22.3 (93)	35.9 (142)	FE, *p* <.001 1 < 2
Gnawing	15.6 (65)	23.1 (90)	FE, *p* =.007 1 < 2
Sharp	39.4 (164)	48.5 (191)	FE, *p* =.011 1 < 2
Tender	41.2 (171)	50.0 (197)	FE, *p* =.013 1 < 2
Burning	17.4 (73)	29.3 (115)	FE, *p* <.001 1 < 2
Exhausting	23.1 (96)	47.1 (184)	FE, *p* <.001 1 < 2
Tiring	46.9 (196)	63.5 (250)	FE, *p* <.001 1 < 2
Penetrating	18.5 (77)	33.2 (128)	FE, *p* <.001 1 < 2
Nagging	36.9 (153)	52.3 (205)	FE, *p* <.001 1 < 2
Numb	28.3 (118)	32.7 (128)	FE, *p* =.194
Miserable	22.6 (95)	42.6 (167)	FE, *p* <.001 1 < 2
Unbearable	7.2 (30)	17.6 (69)	FE, *p* <.001 1 < 2

^a^For this class of patients, the overall pain (approximately > 7.0) and sleep disturbance (approximately > 65) scores exceeded the clinically meaningful cutoff and were in the severe range

Abbreviations: *FE*, Fisher’s exact test; *SD*, standard deviation; *U*, Mann–Whitney *U* test

### Sleep disturbance characteristics

Compared to the Both Moderate class, patients in the Both Severe class reported higher scores for all of the GSDS subscales, as well as the total GSDS score (Table 4).

**Table 4:** Differences in sleep characteristics between the joint pain and sleep disturbance classes at enrollment

Sleep disturbance subscales^a^	Moderate pain and sleep disturbance 53.4% (*n* = 519)	Severe pain and sleep disturbance 46.6% (*n* = 453)	Statistics
	Mean (SD)	Mean (SD)	
Quality of sleep (≥ 3.0)	2.5 (1.5)	4.6 (1.4)	*t* = − 21.63, *p* <.001 1 < 2
Quantity of sleep (≥ 3.0)	4.1 (1.5)	5.4 (1.6)	*t* = − 13.17, *p* <.001 1 < 2
Sleep onset latency (≥ 3.0)	1.9 (1.9)	4.2 (2.2)	*t* = − 17.18, *p* <.001 1 < 2
Mid-sleep awakenings (≥ 3.0)	4.3 (2.4)	5.8 (1.6)	*t* = − 12.29, *p* <.001 1 < 2
Early awakenings (≥ 3.0)	2.7 (2.3)	5.0 (2.1)	*t* = − 15.87, *p* <.001 1 < 2
Medications for sleep (≥ 3.0)	0.40 (0.58)	0.98 (0.92)	*t* = − 11.32, *p* <.001 1 < 2
Excessive daytime sleepiness (≥ 3.0)	2.1 (1.2)	3.6 (1.3)	*t* = − 18.13, *p* <.001 1 < 2
GSDS total score (≥ 43.0)	41.6 (14.3)	70.3 (14.5)	*t* = − 30.33, *p* <.001 1 < 2

Abbreviations: *GSDS*, General Sleep Disturbance Scale; *SD*, standard deviation

^a^Clinically meaningful cutoff scores

## Discussion

This study is the first to use LPA to identify distinct joint profiles of pain AND sleep disturbance in oncology outpatients receiving chemotherapy. Almost 50% of the patients had pain scores of approximately seven (i.e., severe pain) [33] and sleep disturbance scores of approximately 70 (i.e., clinically meaningful level of sleep disturbance) [34]. While no studies have done a joint LPA in patients receiving chemotherapy, the prevalence of severe pain in this sample is higher than the 30.6% reported in a meta-analysis of cancer patients undergoing active treatment [1]. In terms of sleep disturbance, our prevalence rate is consistent with the 60% reported in a review of patients with cancer [5]. It should be noted that the GSDS scores of the patients in the Both Severe class are comparable to mothers of newborn infants [34].

It is interesting to note that compared to the LPAs for the single symptoms of pain [4] and sleep disturbance [18], which each found three classes, the current joint analysis identified only two distinct profiles. While both of the single symptom LPAs identified a Low class [4, 18], these same patients in the joint analysis were categorized into one of two profiles in which both symptoms were of a moderate or severe intensity. As shown in Table 5, one in five patients from the Low Pain class in the single symptom LPA [4] shifted to the Both Severe class in the joint LPA and one in three patients from the Moderate Pain class shifted to the Both Severe class. In terms of sleep disturbance, 100% of the patients in the Low Sleep Disturbance class in the single symptom LPA [18] were classified into the Both Moderate class in the joint LPA and 99.6% of the patients in the Very High Sleep Disturbance class were classified into the Both Severe class (Table 6). These findings suggest that interaction effects occur between these two symptoms when they co-occur in the same patient. Future studies need to confirm these findings and determine which symptom is driving the co-occurrence and severity of the other symptom. This information is important in planning interventions to prevent or mitigate both pain and sleep disturbance.

**Table 5:** Differences in class membership between the worst pain LPA versus the joint LPA for worst pain and sleep disturbance

Joint Worst Pain AND Sleep Disturbance classes	Only Worst Pain classes			
	Low pain % (*n*)	Moderate pain % (*n*)	Severe pain % (*n*)	Worst Pain AND Sleep Disturbances totals
Both Moderate	79.8 (130)	63.3 (236)	33.9 (135)	501
Both Severe	20.2 (33)	36.7 (137)	66.1 (263)	433
Only Worst Pain totals	163	373	398	

Abbreviation: *LPA*, latent profile analysis

Shin, J., Oppegaard, K., Calvo-Schimmel, A., Harris, C., Cooper, B. A., Paul, S. M., Conley, Y. P., Hammer, M. J., Cartwright, F., Kober, K. M., Levine, J. D., & Miaskowski, C. (2023). Distinct worst pain profiles in oncology outpatients undergoing chemotherapy. *Cancer Nursing*, *46*(3), 176

**Table 6:** Differences in class membership between the sleep disturbance LPA versus the joint LPA for worst pain and sleep disturbance

Joint Worst Pain AND Sleep Disturbance classes	Only Sleep Disturbance classes			
	Low sleep disturbance % (*n*)	High sleep disturbance % (*n*)	Very high sleep disturbance % (*n*)	Worst Pain AND Sleep Disturbance totals
Both Moderate	100.0 (193)	65.2 (306)	0.4 (1)	500
Both Severe	0.0 (0)	34.8 (163)	99.6 (268)	431
Only Sleep Disturbance totals	193	469	269	

Abbreviation: *LPA*, latent profile analysis, Tejada, M., Viele, C., Kober, K. M., Cooper, B. A., Paul, S. M., Dunn, L. B., Hammer, M. J., Wright, F., Conley, Y. P., Levine, J. D., & Miaskowski, C. (2019). Identification of subgroups of chemotherapy patients with distinct sleep disturbance profiles and associated co-occurring symptoms. *Sleep*, 42(10), zsz151

The next sections of the Discussion compare the risk factors associated with membership in the Severe Worst Pain [4] and Very High Sleep Disturbance [18] classes from the single symptom analyses to those associated with the Both Severe class from the joint LPA (Table 7). This type of evaluation can assist clinicians to identify patients at increased risk for high levels of one or both symptoms and prescribe interventions for modifiable risk factors.

**Table 7:** Characteristics associated with membership in the Severe Worst Pain class and the Very High Sleep Disturbance class compared to the Severe Pain AND Sleep Disturbance class

Characteristics	Single symptom LPA^1^	Single symptom LPA^2^	Joint LPA
	Severe Worst Pain^a^	Very High Sleep Disturbance^b^	Severe Pain and Sleep Disturbance^c^
Demographic characteristics			
Younger age		■	■
Fewer years of education	■		■
More likely to be female	■	■	■
Less likely to be Asian or Pacific Islander			■
More likely to be Hispanic, Mixed race, or other			■
Less likely to be married or partnered	■	■	■
More likely to live alone		■	■
Less likely to be currently employed	■	■	■
More likely to have a lower annual household income	■	■	■
More likely to have child care responsibilities		■	■
Less likely to exercise on a regular basis		■	■
Clinical characteristics			
Higher body mass index		■	■
Lower Karnofsky Performance Status score	■	■	■
Higher number of comorbid conditions	■	●	■
Higher Self-administered Comorbidity Questionnaire score	■	■	■
Higher MAX2 score	■		
Less likely to have gastrointestinal cancer	■	■	
More likely to report ulcer or stomach disease		●	
More likely to self-report anemia or blood disease	■	●	
More likely to self-report depression	■	●	■
More likely to self-report osteoarthritis	■	●	
More likely to self-report back pain	■	●	■
More likely to have received only CTX		●	■
Less likely to have received both CTX and targeted therapy		●	■

● Results not reported in original publication

■ Indicates significant differences between the classes

Abbreviation: *CTX*, chemotherapy

^a^Comparisons done between the Low Pain and the Severe Pain classes

^b^Comparisons done between the Low Sleep Disturbance and the Very High Sleep Disturbance classes

^c^Comparisons done between the Moderate Pain AND Sleep Disturbance and the Severe Pain AND Sleep Disturbance classes

^1^Shin, J., Oppegaard, K., Calvo-Schimmel, A., Harris, C., Cooper, B. A., Paul, S. M., Conley, Y. P., Hammer, M. J., Cartwright, F., Kober, K. M., Levine, J. D., & Miaskowski, C. (2023). Distinct worst pain profiles in oncology outpatients undergoing chemotherapy. *Cancer Nursing*, *46*(3), 176

^2^Tejada, M., Viele, C., Kober, K. M., Cooper, B. A., Paul, S. M., Dunn, L. B., Hammer, M. J., Wright, F., Conley, Y. P., Levine, J. D., & Miaskowski, C. (2019). Identification of subgroups of chemotherapy patients with distinct sleep disturbance profiles and associated co-occurring symptoms. *Sleep*, 42(10), zsz151

### Common demographic risk factors

Across the three LPAs, the common demographic risk factors were being female, single, unemployed, and reporting a lower annual household income. These findings are consistent with studies from the general population, which demonstrated associations between higher levels of pain and/or sleep disturbance and female gender [35, 36], being unmarried [37, 38], being unemployed [39, 40], and having a lower income [38, 39]. In terms of pain, socioeconomic disadvantage is a significant predictor of disabling pain in the general population [41, 42]. Equally important, patients experience significant financial toxicity associated with cancer and its treatments. As noted in one review [43], risk factors associated with increased financial toxicity in these patients include belonging to a racial/ethnic minority group, being unmarried or partnered, living in a rural area, having a lower household income, and being uninsured. While insurance status was not evaluated in the current study, an analysis from the 2022 National Health Survey found that among US adults who were younger than 65 years, being unmarried, unemployed, and having a lower income were associated with a lack of health insurance. In addition, financial toxicity is associated with a higher overall symptom burden in patients undergoing cancer treatment [44].

In terms of sleep disturbance, evidence from a meta-analysis suggests that worldwide, women report higher rates of sleep disturbance that increases with age [36]. Potential explanations for this finding include hormonal variations associated with menopause [45] and higher rates of anxiety and depression in women [46]. However, in a study from the UK [38], the authors argue against the latter hypothesis. Findings from their study suggest that socioeconomic factors explain over half of the gender differences in sleep disturbance and that adjusting for depression and health status widens the gender gap. The authors caution sleep researchers not to conflate “worries” that reflect a rational reaction to difficult socioeconomic circumstances with an underlying mental illness. They noted that interventions to address each of these risk factors, if available, are distinct. Additional research is warranted on the impact of socioeconomic disadvantage and financial toxicity on both pain and sleep disturbance in patients with cancer.

### Unique demographic risk factors

Younger age, living alone, having child care responsibilities, and lack of regular exercise were associated with membership in both the Very High Sleep Disturbance class and the Both Severe Class (see Table 7). While in the general population, pain intensity and interference tend to increase with age [41, 47], older oncology patients tend to report lower occurrence rates for and levels of pain [48, 49]. In terms of age differences in sleep disturbance, findings are inconsistent. As noted in a review of sleep difficulties in adults in the USA [50], while younger individuals tend to report more trouble falling asleep, older people have more trouble staying asleep. However, in a longitudinal study of adults in the UK [51], while no associations were found between age and the incidence of insomnia, older age was identified as a risk factor for persistent insomnia at 1 year after enrollment.

While reasons for these age differences are not readily apparent, in a study that evaluated the relationships between sleep disturbance, depressed mood, pain, and fatigue [52], the authors suggested that because younger patients with a higher functional status receive more aggressive chemotherapy, they experience a higher symptom burden. However, in the current study, while patients in the Both Severe class were younger, MAX2 scores (a measure of treatment toxicity) for the Both Moderate and Both Severe classes were similar. In addition, given that patients in the Both Severe class were more likely to have received only chemotherapy and patients in the Both Moderate class were more likely to have received a combination of chemotherapy and a targeted therapy, treatment modality may influence the co-occurrence and severity of comorbid pain and sleep disturbance. Additional research is warranted on age-related differences in the causes and severity of pain and sleep disturbance in patients undergoing various types of cancer treatment.

Evidence on the relationship between living alone and pain and/or sleep disturbance is inconsistent. For example, several studies documented poorer outcomes in patients who lived alone (e.g., longer time to diagnosis [53]; lower rates of adherence with endocrine therapy [54]; increased risk for the development of peripheral neuropathy [55]; increased odds of not receiving guideline-recommended treatment [56]) that could be associated with a higher symptom burden. In contrast, in two prospective studies [57, 58], no differences in cancer treatment outcomes were found between patients who did and did not live alone. Additional research is warranted to better understand how patients’ living arrangements mediate and/or moderate associations between pain and/or sleep disturbance. Potentially confounding factors that deserve particular attention include transportation barriers (e.g., distance between home and the clinic), degree of social isolation, and level of dependency (frailty).

While the positive effects of exercise on fatigue severity are well documented [59, 60], emerging evidence suggests that this intervention is effective for some types of cancer pain. For example, the results of a scoping review suggest that adapted physical activity programs can reduce joint pain in breast cancer patients who are taking aromatase inhibitors [61]. In another review that evaluated the effects of nineteen exercise interventions on pain and balance in patients with chemotherapy-induced peripheral neuropathy [62], the most effective interventions for pain included hand-foot exercises; aerobic exercise combined with resistance training; and muscular strength combined with balance exercises. In another systematic review that compared the effects of different exercise interventions on cancer pain [63], a comprehensive exercise program (i.e., a program that included aerobic exercise, resistance exercise, flexibility exercise, and a plan for the intensity and duration of the exercise) was more effective than usual care in decreasing pain intensity. Given that 35.7% and 52.1% of the patients in the Both Moderate and Both High classes, respectively, reported both cancer and non-cancer pain, additional research is warranted on the efficacy of different types of exercise interventions for different types of pain in these patients.

In terms of the effects of exercise on sleep disturbance in patients with cancer, several systematic reviews reported positive findings [64–66]. For example, in a review that evaluated the effects of resistance training [64], the meta-analytic findings suggest that combined aerobic and resistance training resulted in significant improvements in sleep quality and insomnia. In another review that evaluated the efficacy of exercise interventions for cancer patients with insomnia [65], the authors concluded that a substantial amount of heterogeneity existed across the nine studies evaluated; findings were suggestive of the benefits of exercise; and additional research was warranted to develop individualized exercise prescriptions. In the most recent review that evaluated the efficacy of pharmacologic and nonpharmacologic interventions for insomnia in cancer patients [66], while exercise showed some benefits, it was not as efficacious as cognitive behavioral therapy for insomnia. Within the context of the current study, future research needs to evaluate the efficacy of exercise interventions in oncology patients who have both pain and sleep disturbance.

### Common clinical risk factors

Across the three LPAs, the common clinical risk factors were a lower functional status, a higher number of comorbid conditions, a higher comorbidity burden, and higher self-reported occurrence rates for depression and back pain. In the current study, the difference in KPS scores between the Both Moderate and Both Severe classes represents not only a statistically significant, but clinically meaningful differences in functional status (81.1 ± 11.9 vs. 74.7 ± 12.1, Cohen’s *d* = 0.52 [67]) [68, 69]. This finding is consistent with previous studies of both the general population [70, 71] and patients with cancer [15, 72].

Comorbidities are common in cancer patients who report pain and sleep disturbance [11, 73]. Consistent with previous reports in both the general population [73] and cancer patients [40], a variety of chronic conditions (e.g., congestive heart failure, obstructive airway disease, musculoskeletal conditions) are associated with increases both pain and sleep disturbance.

Self-reported diagnoses of depression and back pain were common characteristics associated with membership in the worst symptom profiles for all three LPAs (i.e., the single symptoms of pain [4] and sleep disturbance [18] and the joint LPA of pain and sleep disturbance). In the general population, depression occurs in 39.3% of patients with chronic pain [74] and in 20% of patients with insomnia [75]. In a recent review of depression in cancer patients [76], the global prevalence rate of 33.2% is comparable to the rate reported by patients in the Both Severe class (i.e., 31.6%). In one longitudinal study of oncology patients with pain and/or depression, improvement in depression was a strong predictor of subsequent improvement in pain [77]. In another longitudinal study of patients with non-cancer pain [78], a strong and equally bidirectional relationship was found between pain and depression. The relationships among depression, chronic pain, and sleep disturbance can be partially explained by shared biological mechanisms. As noted in a recent review [79], evidence suggests that alterations in brain structures (e.g., atrophy of the hippocampus), as well as changes in neural pathways (i.e., increased sensitization of the central nervous system) and various biological processes (e.g., inflammation; alterations in monoamine neurotransmitters, alterations in brain derived neurotropic factor), underlie these three symptoms. Equally important, evidence suggests that individuals who develop one of these three symptoms are at increased risk for developing the other two [81].

While low back pain is a common global problem with a point prevalence rate of 7.5% [80], 28.3% and 31.6% of the patients in the Both Moderate and Both Severe classes, respectively, reported this comorbidity using the SCQ. However, it is worth noting that on the questionnaire that evaluated causes of non-cancer pain (Table 3), the reported percentages were higher (i.e., 38.7% and 41.2%). In addition, while not evaluated on the SCQ, 26.9% and 41.2% of the patients in the Both Moderate and Both Severe classes, respectively, reported headaches. Given that the etiologies for these two pain conditions were not evaluated in the current study, their high occurrence rates suggest that clinicians need to obtain more detailed information and initiate referrals and/or interventions as warranted by the findings. In terms of associations between back pain and sleep disturbance, findings from a systematic review and meta-analysis suggest that in patients with chronic spinal pain, higher rates of insomnia were associated with the presence of severe pain, depression, and anxiety [81]. Future studies need to determine the underlying mechanisms for these common chronic conditions.

### Pain characteristics

As shown in Table 3, the current study compared the Both Moderate and Both Severe classes on a number of pain characteristics (e.g., intensity, qualities, interference). It should be noted that for all of the characteristics, except the quality of numbness, patients in the Both Severe class reported higher scores. Equally important, patients in the Both Severe class reported pain intensity scores in the severe range; that occurred in approximately ten different locations; and persisted for 10 h per day. In addition, all of the interference scores were in the moderate range (i.e., ≥ 4.0) with the highest scores reported for interference with mood, normal work, sleep, enjoyment of life, and sexual activity.

In terms of the causes of pain, while no comparative studies were identified, over 30% of the patients in both classes reported only non-cancer pain. However, 36% and 52% of the patients in the Both Moderate and Both Severe classes, respectively, reported the occurrence of both cancer and non-cancer pain. While the causes of cancer pain were not evaluated in the current study, the most common causes of non-cancer pain across the total sample were low back pain (44.7%), headache (34.3%), and arthritis (26.5%). Of note, 59.1% and 72.7% of the patients in the Both Moderate and Both Severe classes, respectively, reported that their non-cancer pain persisted for greater than 6 months. In contrast, the 6-month duration of cancer pain was reported by only 23.8% and 27.1% of the Both Moderate and Both Severe classes, respectively. While additional studies are warranted on the additive and/or synergistic effects of single versus multiple causes of chronic pain in oncology patients [82], these findings suggest that oncology and primary care clinicians need to collaborate to optimize pain management across both types of pain.

Given the original purposes of the current study, a detailed characterization of the pharmacologic and non-pharmacologic interventions that these patients used was not done. However, 54.4% and 71.7% of the patients in the Both Moderate and Both Severe classes, respectively, reported using analgesics in the past week. It is interesting to note that while both classes reported comparable ratings for percentage of pain relief from pain medication (i.e., 67.4% and 67.5%), the worst pain score for the Both Severe class at enrollment was statistically significantly higher and as expected demonstrated a clinically meaningful difference (i.e., Cohen’s *d* =  − 0.60 [67]) [68, 69]. In terms of satisfaction with pain management, while the Both Severe class reported significantly lower scores (i.e., 6.9 versus 7.5 for the Both Moderate class), the effect size for the between group differences was relatively small (i.e., Cohen’s *d* = 0.24). This mismatch between high pain intensity scores and high satisfaction scores is consistent with previous studies of postoperative pain [83, 84]. Qualitative studies are needed to explore these “mismatched” findings.

While not exhaustive in its evaluation of pain and pain management, the findings presented in Table 4 suggest some testable hypotheses. For example, given that the patients in the Both Severe class were slightly younger, they may have had an increased sensitivity to pain (i.e., lower pain threshold) [85]. In addition, given the high percentages of patients with both cancer and non-cancer pain of greater than 6-month duration, these patients may be experiencing central sensitization [86]. Equally important, a relatively high percentage of patients had comorbid conditions that may contribute to central sensitization and what Mayer and colleagues refer to as a “central sensitivity syndrome” [87].

### Sleep characteristics

While the total GSDS scores for the Both Moderate class hovered at the clinically meaningful cutoff of 43.0 over the six assessments, the Both Severe class’s scores were in the 70 s over the 2 months of the study. This finding suggests additive or synergistic effects between pain and sleep disturbance in oncology patients receiving chemotherapy.

The trajectories of sleep disturbance differed between the Both Moderate and Both Severe classes (Fig. 1). While slight increases in GSDS scores are apparent in the Both Severe class at the second and fifth assessments (i.e., following chemotherapy administration), these increases were more pronounced in the Both Moderate class. These findings are consistent with a previous study of patients with breast cancer [88] that found subgroups of women with distinct sleep disturbance trajectories during chemotherapy. Possible explanations for the distinct trajectories include as follows: inadequate management of nausea and vomiting in the Both Moderate class; pre-existing insomnia or obstructive sleep apnea in the Both Severe class; and/or ineffective pain management in the Both Severe class.

In terms of the GSDS subscales, patients in the Both Severe class reported significantly higher scores for all seven subscales (Table 4). In the Both Moderate class, only two subscales (i.e., quantity of sleep (with higher scores indicating an insufficient amount of sleep); mid-sleep awakenings) exceed the clinically meaningful cutoff score. This finding suggests that these patients may be experiencing problems with sleep maintenance. In contrast, in the Both Severe class, except for use of medications for sleep, all of the other GSDS subscale scores exceeded the clinically meaningful cutoff. Given that these patients reported difficulties with falling asleep (i.e., sleep onset latency) and staying asleep (i.e., mid-sleep awakenings, early awakenings) suggests that they had problems with both sleep initiation and maintenance. Of note, and consistent with a previous study [15] and practice guidelines [89], both classes used very little sleep medications. However, patients in the Both Severe class used sleep aids about twice as often as the Both Moderate class, a trend which was found to be associated with higher levels of pain and depression [90].

### Relationships between pain and sleep disturbance

Undoubtedly, the relationships between pain and sleep disturbance are extremely complex. For example, in a study of healthy pain free adults, shortened sleep duration was associated with lower pain thresholds the following day [91]. In patients with cancer [92–94], inadequate and/or low-quality sleep prior to surgery or radiotherapy was associated with increased pain severity and/or pain interference following the intervention. However, evidence for this kind of temporal relationship between pain and sleep disturbance is less definitive in patients with chronic pain [95, 96]. Given the relatively stable and high levels of pain and sleep disturbance in the Both Severe class, additional longitudinal studies are needed that examine the relationships between acute and chronic pain and various aspects of sleep disturbance in oncology patients with different types of pain and/or sleep disturbance. In addition, given the high prevalence of depression in the Both Severe class, future studies should evaluate latent variable models of pain AND depression and sleep disturbance AND depression. Equally important, given that numerous mechanisms are proposed to explain the relationships between pain and sleep disturbance (e.g., alterations in the hypothalamic pituitary adrenal axis [97]; dysregulation of the circadian clock [98]; neuroinflammation [99]), future longitudinal studies should collect biospecimens so that various underlying mechanisms can be evaluated.

### Limitations

Several limitations warrant consideration. The sample was relatively homogeneous in terms of gender, education, and self-reported ethnicity. Given that previous studies found ethnic differences in sleep disturbance [100–102] and pain [103, 104], future studies need to recruit a more diverse sample of patients and obtain data on additional social determinants of health. Patients were not recruited prior to the initiation of chemotherapy or followed through to the completion of treatment. Prospective longitudinal studies need to evaluate the relationships between pain, sleep disturbance, and other common symptoms during the entire treatment period and into survivorship. Equally important, more detailed histories of pain (e.g., specific causes of both cancer and non-cancer pain), sleep disturbance (e.g., history of obstructive sleep apnea), and other symptoms (e.g., depression, nocturia, hot flashes) are warranted in future studies. Because this study used only a subjective measure of sleep disturbance, an objective sleep measure (e.g., actigraphy) needs to be added in future studies. Given the discordance between subjective and objective measures of sleep disturbance [105], comparisons of the findings from latent variable models that use subjective and objective measures of sleep, as well as biomarkers, may increase our knowledge of risk factors and underlying mechanisms.

### Implications for clinical practice

The findings from this study demonstrate that a significant proportion of patients receiving chemotherapy experience the co-occurrence of severe pain and sleep disturbance. Therefore, clinicians need to perform routine assessments for both pain and sleep disturbance. In addition, they can use the list of modifiable and non-modifiable risk factors found in this study to identify high-risk patients. The findings suggest that some patients warrant referrals to social services. Equally important, given the number of chronic painful conditions reported by the patients in the Both Severe class, oncology clinicians need to work with primary care providers to optimize the management of these conditions and provide effective pain management.

Given that pain and sleep disturbances have reciprocal relationships (i.e., the worsening of either symptom may exacerbate the other), a vicious cycle can occur with deleterious consequences. Patients with either or both symptoms should be counseled on the importance of sleep hygiene (i.e., the habits and environment that promote healthy restful sleep) and physical activity. In addition, clinicians should consider referrals for physical therapy, cognitive behavioral therapy for insomnia or pain, and other non-pharmacologic interventions to decrease patients’ symptom burden.

## Data Availability

Data is available from the corresponding author after the completion of a data transfer agreement with the University of California, San Francisco.
